# Family Cohesion Is Associated with the Self-Perceived Need for Dental Treatment among Adolescents

**DOI:** 10.1155/2021/4504030

**Published:** 2021-09-29

**Authors:** Isolda M. L. F. Prata, Ana Flávia Granville-Garcia, Érick T. B. Neves, Larissa C. M. Lima, Laio C. Dutra, Matheus F. Perazzo, Fernanda M. Ferreira, Saul M. Paiva

**Affiliations:** ^1^Department of Dentistry, School of Dentistry, State University of Paraíba, Campina Grande 58429-500, Brazil; ^2^Department of Paediatric Dentistry, School of Dentistry, Federal University of Minas Gerais, Minas Gerais 31270-901, Brazil

## Abstract

This study explored the association between family cohesion and self-perceived need for dental treatment among adolescents. A school-based representative cross-sectional study was conducted with 746 students aged 15 to 19 randomly selected from schools in Campina Grande, Brazil. Parents/guardians provided information on sociodemographic data, and students completed questionnaires about the self-perceived need for dental treatment, dental pain, and family cohesion and adaptability (FACES III). Two dentists were trained (kappa >0.80) to diagnosis dental caries using the Nyvad criteria and assess adolescents' level of functional oral health literacy (BREALD-30). Descriptive analysis was performed, followed by nonadjusted and adjusted robust binary logistic regression for complex samples (*α* = 5%). The prevalence of self-perceived need for dental treatment was 88.6%. The presence of dental caries (OR = 2.10; IC 95%: 1.22–3.61), tooth loss (OR = 15.81; IC 95%: 2.14–116.56), dental pain in the last six months (OR = 1.87; IC 95%: 1.06–3.31), and enmeshed family cohesion type (OR = 10.23; IC 95%: 3.96–26.4) remained associated with the self-perceived need for dental treatment in the final model. In conclusion, dental caries, dental pain, tooth loss, and family cohesion influenced the self-perceived need for dental treatment in adolescents.

## 1. Introduction

Self-perceived need for dental treatment is a subjective measure that depends on how people assess their oral health condition [1–3] and may influence the search for dental services [4], acting as an important predictor of dental care utilization [5]. Studies on the self-perceived need for dental treatment have been conducted mainly in adults and the elderly [2, 6, 7]; however, adolescence is considered a strategic period for such studies due to the large number of changes that occur in this phase and because oral habits formed in adolescence may persist later in adulthood [8, 9]. Also, oral hygiene practices are often neglected during adolescence and this group is exposed to risk factors for oral diseases such as unbalanced sugar intake and a low frequency of dental visits [10–12]. Moreover, a previous study conducted in Brazil among adolescents demonstrated that the self-perceived need for dental treatment was the main reason for dental visits for this age group [13].

The knowledge regarding the factors associated with the self-perceived need for dental treatment in adolescents can assist the planning of education, prevention, and health promotion programs for this target population. Previous studies have shown that aspects such as lower quality of life related to oral health, sociodemographic factors, searching dental services for treatment, dental pain, dissatisfaction with teeth and mouth, and dental caries were associated with the self-perceived need for dental treatment in adolescents [3, 14]. Another factor that has been associated with oral health outcomes in adolescents is oral health literacy (OHL) [15, 16], which is the ability to identify, understand. and apply oral health information and influences health decision-making [17, 18]. Thus, it is important to evaluate the influence of this social determinant of health on the self-perceived need for dental treatment given that there is no previous study conducted in this perspective.

Adolescents are commonly dependent on family members, hence are influenced by the family environment [19, 20]. Therefore, another important aspect that should be considered during adolescence is related to the family environment. Family cohesion is a component of family functioning and assesses the level of proximity among family members [21]. A previous study showed that higher levels of family cohesion can positively contribute to adequate health behaviors in adolescence [22], with low levels of family cohesion contributing to lower life satisfaction in adolescents [23], probably reducing motivation and interest in issues related to oral health. A growing number of cross-sectional studies have examined the association between low family cohesion and dental problems such as dental caries in adolescents [16, 24] but the role of family cohesion in adolescents' self-perceived need for dental treatment has not been explored. This information is important because adolescents are influenced by the family environment to make decisions, as well as establish financial, psychological, and emotional dependence with guardians and parents [20, 25, 26].

The conceptual hypothesis of this study was that a higher level of family cohesion influences the prevalence of self-perceived need for dental treatment in adolescents; thus, this study assessed the association between family cohesion and self-perceived need for dental treatment in adolescents aged 15 to 19.

## 2. Materials and Methods

### 2.1. Ethical Issues

This study was approved by the Human Research Ethics Committee of the State University of Paraiba (certificate number: 55953516.2.1001.5187) and followed the guidelines established by the Declaration of Helsinki. Parents/caregivers signed an informed consent form, and adolescents signed a statement of informed consent authorizing their participation in the research.

### 2.2. Study Design and Sample Selection

An analytical, cross-sectional, school-based study was conducted with adolescents aged 15 to 19 years enrolled in public and private schools in a city in Campina Grande, Brazil. Data was collected between October 2016 and July 2017. Students undergoing orthodontic treatment, with learning problems, neurological disorders and/or physical disabilities, or in need of specialized support were excluded from the study. Probabilistic sampling by clusters was conducted in two stages. In the first stage, 16 public schools and 16 private schools were randomly selected in the city's six administrative districts. In the second stage, students were selected by simple random sampling in each school.

The sample calculation was performed for analytical comparative studies between two independent proportions using Software G^∗^Power version 3.1 (Franz Faul, Universitat Kiel, Germany) adopting a significance level of 95% and power of 80%. The proportion estimates of the pilot study indicated a prevalence of self-perceived need for dental treatment in individuals from disconnected and bonded families of 76.4% and 87.5%, respectively. The minimum sample calculated was 376, to which a design effect of 1.6 was applied, reaching a sample of 602. To this number, 20% was added to compensate for possible losses, reaching a final sample of 753 participants.

### 2.3. Training and Calibration Exercises

Two dentists were trained to diagnose dental caries using the criteria proposed by Nyvad and Baelum [27] by an expert in the field of diagnosis and epidemiology and took place in two stages according to the method proposed by Peres et al. [28]. The first theoretical stage involved the study of the diagnostic criteria through the projection of images of the conditions that could be observed in the clinical examination, clinical records, and the steps to follow in the clinical examination. In the practical stage, gold standard clinical examinations were conducted by the examiners of fifty adolescents aged 15 to 19 years old from a public school. The students were reexamined after an interval of seven days to determine the interexaminer (kappa = 0.89 to 0.90) and intraexaminer (kappa = 0.88 to 0.90) agreement. Adolescents examined in the calibration exercise were not included in the main study.

Two interviewers were trained and calibrated to apply the Brazilian version of the Rapid Estimate of Adult Literacy in Dentistry (BREALD-30) following the methodology of Vilella et al. [29]. A researcher considered the gold standard in OHL, and BREALD-30 was responsible for this step. Theoretical training was first carried out on the application criteria of the instrument; then, practical training and calibration were performed using a bank of 15 videos of individuals with different levels of OHL. The agreement between the two examiners was 0.870. and the intraexaminer agreement was 0.898 and 0.871. Kappa coefficients between examiners and the gold standard were 0.889 and 0.884.

### 2.4. Pilot Study

To test and evaluate the proposed methodology, a pilot study was conducted with fifty adolescents aged 15 to 19 years randomly selected from a private school (*n* = 25) and a public school (*n* = 25) and was not included in the main sample. The results of this stage did not reveal the need for changes to the study methodology.

### 2.5. Nonclinical Data Collection

The parents/caregivers completed a sociodemographic questionnaire collecting information about the adolescent's sex, self-declared color, and maternal education. To determine the social class of the adolescents, the Brazil Economic Classification Criterion proposed by the Brazilian Association of Research Companies was used, which considers the education of the head of the family, the number of consumer goods reported by parents/guardians, and access to public services [30]. Scores were attributed to each item, and the total sum allows families to be classified in decreasing order of economic favoring in classes A, B1, B2, C1, C2, D, and E. For the present study, the social class was dichotomized upwards (classes A and B) or low (classes C, D, and E) [21]. The adolescents were required to complete a questionnaire on Access and Use of Health Services of the National Oral Health Survey to assess the self-perception of the need for treatment and self-report of dental pain in the last six months [31].

The Family Adaptability and Cohesion Evaluation Scales (FACES III) is validated for use in Brazil and was completed by adolescents to assess levels of family cohesion and adaptability. FACES III contains 20 questions, 10 even number questions related to family cohesion, and 10 odd questions address family adaptability. According to the family cohesion scores, families were classified into four groups: enmeshed (high degree of dependency among family members), connected (moderate degree of independence between family members), separated (considerable degree of independence between family members), and disengaged (very high degree of independence among family members). The level of family adaptability was classified as very flexible/chaotic (very high family adaptability), flexible (moderate to high family adaptability), structured (low to moderate family adaptability), and rigid (very low family adaptability) [32].

BREALD-30 was applied to measure the functional OHL of adolescents, which was validated for use with this population [33]. This instrument contains thirty words related to dentistry following an increasing order of reading difficulty. The students read the words out loud to the examiner, and a point is given for each word pronounced correctly and zero for incorrect pronunciation; thus, higher scores denote a higher level of OHL. The total BREALD-30 score was categorized into inadequate, marginal, or adequate literacy based on the distribution of scores, 0–18, 19–22, and 23–30, respectively, using sample distribution tertiles as cutoff points.

### 2.6. Clinical Data Collection

Prior to the clinical examination, the adolescents underwent supervised brushing and topical application of fluoride. In a private room at the school, students were examined individually, sitting in a school chair in front of the examiner. The participants' teeth were evaluated with the aid of a headlight (Petzl Zoom headlamp, Petzl America, Clearfield, UT, USA), mouth mirror (PRISMA®, São Paulo, SP, Brazil), and previously sterilized WHO periodontal probe (GOLGRAN®, São Paulo, SP, Brazil). To dry the dental surfaces before the clinical examination, sterile gases were used, as well as relative insulation with a cotton roll to keep the teeth dry. The biosafety protocols were followed by the examiners who used the necessary personal protective equipment.

Dental caries was diagnosed using the Nyvad Classification [27] based on visual and tactile findings and allows to detect the activity and severity of injuries. In the present study, the codes considered to demonstrate the presence of caries were 2 (active caries with a discontinuous surface), 3 (active caries with cavitation), 5 (inactive caries with a discontinued surface), and 6 (inactive caries with cavitation). Tooth loss due to caries was assessed during the oral clinical examination.

### 2.7. Statistical Approach

Descriptive analysis was performed, followed by unadjusted and adjusted analyzes. The dependent variable was the self-perceived need for treatment (yes and no), and the independent variables were sociodemographic characteristics, OHL, dental caries, presence of toothache in the last 6 months, and loss of permanent teeth due to caries, family cohesion, and family adaptability. Associations between independent variables and self-perceived need for treatment were tested by robust logistic regression for complex samples. Variables with a *p* value <0.20 in the unadjusted analysis were incorporated into the adjusted analysis, and those with a *p* value <0.05 after the adjustments were considered significantly associated with the outcome. Data organization and statistical analysis were performed using the SPSS (SPSS for Windows, version 22.0; IBM Inc., Armonk, NY, USA).

## 3. Results

Seven hundred and forty-six adolescents aged 15 to 19 years participated in the study. The losses of seven students occurred due to their absence on two consecutive days after the exams. The prevalence of adolescents who perceived the need for treatment was 88.6%.  Table 1 presents the subjects' demographic characteristics, showing that most adolescents were female and 71.7% declared themselves to be non-White and low social class (57.4%), and mothers had 8 or more years of schooling (59.7%). More than a third of the adolescents had a marginal level of OHL (37.5%). Family cohesion of the disengaged type (46.1%) and family adaptability of the flexible type (32.8%) were most common.

In the adjusted analysis (Table 2), the variables that remained associated with the self-perceived need for dental treatment were the presence of dental caries (OR = 2.10; 95% CI: 1.22–3.61), loss of permanent teeth due to caries (OR = 15.81; 95% CI: 2.14–116.56), the presence of toothache in the last 6 months (OR = 1.87; 95% CI: 1.06–3.31), and family cohesion of the agglutinated type (OR = 10.23; 95% CI: 3.96–26.4).

## 4. Discussion

The conceptual hypothesis of this study was confirmed that adolescents from enmeshed families and those with dental caries, tooth pain, and tooth loss demonstrated a higher prevalence of self-perceived need for dental treatment compared to those who did not present these conditions. The prevalence of self-perceived need for dental treatment in this study was high (88.6%), higher than that reported (62.6%) in a previous study conducted with Brazilian adolescents from the southeast region [14]. These differences may be related to regional and socioeconomic discrepancies in Brazil as this study was conducted in a city located in the northeast, a region that exhibits a higher prevalence of oral health problems compared to the southeast [31].

Dental caries was associated with the self-perceived need for dental treatment in adolescents, in line with studies conducted in Brazilian adolescents aged 15–19 [14, 34]. Adolescents from Thailand aged 12–15 who had a higher number of untreated dental caries also demonstrated an increased chance to perceive the need for dental care [3]. Dental caries remains a global public health problem and are associated with a poor quality of life related to oral health and may restrict adolescents from daily normal activities [8, 35]. Therefore, dental caries influence oral health self-perception in adolescence [36], probably because this population has increased interest in the aesthetic [37] aspect to maintain social relationships [11]. This finding is important because untreated dental caries can lead to pain and tooth loss [38]; thus, general society, dentists, and policymakers should implement preventive measures and early treatment for dental caries.

Another condition associated with the self-perceived need for dental treatment was dental pain in the last 6 months. Dental pain is a serious public health problem [39] and is associated with a poorer quality of life [40], oral health dissatisfaction [41], sleep disorders, and limitations to daily school activities among adolescents [1]. It may also lead to discomfort which may influence the self-perceived need for dental treatment. In this sense, a previous study conducted in Brazil with adolescents aged 15–19 demonstrated that dental pain at any time in life was associated with a higher prevalence of dental visits [13], indicating a symptomatic pattern for seeking dental services among adolescents [14]. This could be avoided through oral health education regimens to stimulate preventive dental appointments in addition to dental visits only due to clinical symptoms.

Tooth loss due to untreated dental caries was associated with the self-perceived need for dental treatment, suggesting that tooth loss negatively impacts adolescents' quality of life [35] and impairs basic functions such as chewing, speaking, and self-confidence [42]. Thus, tooth loss may influence the self-perceived need for dental treatment in this population. Although this problem is largely preventable by adequate oral health practices [43, 44], the prevalence of tooth loss increases with aging [45]; therefore, adolescence is a critical period to strengthen preventive measures and implement adequate oral health behaviors contributing to maintaining a better oral health status later in life.

Regarding family cohesion, adolescents who were from enmeshed families (high level of family cohesion) had an increased self-perceived need for dental treatment. A previous study conducted in Brazil showed that adolescents aged 12 who had high family cohesion went to dental visits more frequently [21]. Furthermore, other studies of adolescents revealed that a low level of family cohesion was associated with untreated dental caries [16, 24]. Therefore, adolescents from families less connected may present worse oral health behaviors [22]. Although the 15 to 19 age group has greater autonomy compared to younger children [46], a higher level of family cohesion probably indicates greater family support and attention given to the adolescent's oral health [19, 22]. Adolescents who grow up in a cohesive family likely have greater discernment to perceive changes in oral health affecting the self-perceived need for dental treatment, suggesting the importance of valuing family cohesion for the establishment of critical attitudes and oral self-care by adolescents.

OHL was not associated with the self-perceived need for dental treatment. The inclusion of clinical variables and mediators in the model may have hidden this association. Moreover, it is important to clarify that this study included only a functional health literacy measure. BREALD-30 is considered a screening tool and may not have been sensitive enough in these circumstances; however, it is the only validated tool available to date to assess OHL in Brazilian adolescents [33].

A limitation of this study is its cross-sectional design, which does not allow establishing a causal relationship between exposure and outcome. However, methodological procedures were designed to reduce possible bias and increase the internal and external validity of the findings, for example, steps such as sample size calculation, a pilot study, examiners' training, and the use of validated instruments for adolescents were followed. Moreover, epidemiological studies contribute to advances in the field and subside public health policies.

The results of the present study are important to guide oral health education programs in adolescence. The objectives of the Health at School Program (HSP) can be restructured to encourage adolescents' self-criticism in identifying oral health changes early and seeking dental services. Health at School Program is a strategy to promote general and oral health in Brazilian schools. In addition, including the assessment of the family environment is of great relevance in the expansion of healthy behaviors in adolescents. Dental professionals must improve their approach and intervention for adolescents by integrating subjective information into clinical assessments.

## 5. Conclusions

The presence of dental caries, toothache reported in the last 6 months, tooth loss, and family cohesion of the agglutinated type influenced the self-perception of the need for dental treatment in adolescents aged 15 to 19 years.

## Figures and Tables

**Table 1:** Descriptive statistics of 15-19-year-old adolescents.

Variable	*N* (%)
Adolescent sex	
Female	444 (59.5)
Male	302 (40.5)
Self-declared color	
White	211 (28.3)
Non-White	535 (71.7)
Social class	
C-D-E	428 (57.4)
A-B	318 (42.6)
Mother's schooling	
≥8 years of study	443 (59.7)
<8 years of study	299 (40.3)
Oral health literacy	
Inadequate	247 (33.1)
Marginal	280 (37.5)
Adequate	219 (29.4)
Dental caries	
Present	354 (47.5)
Absent	392 (52.5)
Loss of permanent teeth	
Yes	130 (17.4)
No	616 (82.6)
Presence of toothache in the last 6 months	
Yes	281 (38.1)
No	456 (61.9)
Family cohesion	
Enmeshed	15 (2.0)
Connected	121 (16.2)
Separated	266 (35.7)
Disengaged	344 (46.1)
Family adaptability	
Very flexible/chaotic	163 (21.9)
Flexible	244 (32.8)
Structured	216 (29.0)
Rigid	122 (16.4)
Self-perceived need for dental treatment	
Yes	597 (88.6)
No	77 (11.4)

**Table 2:** Nonadjusted and adjusted robust binary logistic regression for the association between family cohesion and self-perceived need for dental treatment among adolescents.

Variable	Self-perceived need for dental treatment					
	Yes *n* (%)	No *n* (%)	*p* value	Nonadjusted OR (95% CI)	*p* value	Adjusted OR (95% CI)
Adolescent sex						
Female	363 (90.3)	39 (9.7)	0.08	1.44 (0.94-2.19)	—	—
Male	234 (86.0)	38 (14.0)	—	1.00	—	—
Self-declared color						
White	165 (88.7)	432 (11.3)	—	—	—	**—**
Non-White	432 (88.5)	56 (11.5)	—	—	—	—
Social class						
C-D-E	359 (91.1)	35 (8.9)	0.03	1.84 (1.03-3.28)	—	—
A-B	238 (85.0)	42 (15.0)	—	1.00	—	—
Mother's schooling						
≥8 years of study	345 (88.0)	47 (12.0)	—	—	—	—
<8 years of study	250 (89.6)	29 (10.4)	—	—	—	—
Oral health literacy						
Inadequate	207 (88.1)	28 (11.9)	—	—	—	—
Marginal	230 (90.6)	24 (9.4)	—	—	—	—
Adequate	160 (86.5)	25 (13.5)	—	—	—	—
Dental caries						
Present	316 (93.8)	21 (6.2)	<0.001	2.99 (1.77-5.07)	0.007	2.10 (1.22-3.61)
Absent	281 (83.4)	56 (16.6)	—	1.00	—	1.00
Loss of permanent teeth						
Yes	124 (99.2)	1 (0.8)	0.003	20.2 (2.79-147-18)	0.007	15.81 (2.14-116.56)
No	473 (86.2)	76 (13.8)	—	1.00	—	1.00
Presence of toothache in the last 6 months						
Yes	249 (93.3)	18 (6.7)	0.002	2.18 (1.32-3.62)	0.030	1.87 (1.06-3.31)
No	341 (85.3)	59 (15.8)	—	1.00	—	1.00
Family cohesion						
Enmeshed	13 (100.0)	0 (0.0)	<0.001	7.66 (1.61-36.34)	<0.001	10.23 (3.96-26.4)
Connected	94 (89.4)	11 (10.5)	—	—	—	—
Separated	208 (86.3)	33 (13.7)	**—**	—	—	—
Disengaged	282 (89.5)	33 (10.5)	—	1.00	—	1.00
Family adaptability						
Very flexible/chaotic	139 (93.3)	10 (6.7)	—	—	—	**—**
Flexible	191 (86.1)	31 (13.9)	0.04	0.45 (0.21-0.97)	—	—
Structured	171 (86.4)	27 (13.6)	0.03	0.44 (0.21-0.93)	—	—
Rigid	95 (91.3)	9 (8.7)	—	1.00	—	—

## Data Availability

The data used to support the findings of this study are available from the corresponding author upon request.
